# Building a public health workforce in Nigeria through experiential training

**DOI:** 10.11694/pamj.supp.2014.18.1.4920

**Published:** 2014-07-21

**Authors:** Akin Oyemakinde, Patrick Nguku, Rebecca Babirye, Sheba Gitta, Peter Nsubuga, Joseph Nyager, Abdulsalami Nasidi

**Affiliations:** 1 Nigeria Centre for Disease Control (NCDC), Federal Ministry of Health, Abuja, Nigeria; 2 African Field Epidemiology Network (AFENET), Abuja, Nigeria; 3 Global Public Health Solutions, Atlanta, Georgia, USA; 4 Federal Ministry of Agriculture and Rural Development, Abuja Nigeria

**Keywords:** Field epidemiology, experiential training, workforce, NFELTP, competent

## Introduction

A competently trained public health workforce that can operate multidisease surveillance and response systems is required for timely detection and response to public health emergencies. The backbone of any disease control is a robust surveillance system that is interlinked with timely quality response[1, 2]. The traditional approaches of training health care workers particularly public health workers have emphasized knowledge acquisition without commensurate competency acquisition; experiential training on the other hand has been successful in creating and sustaining a skilled workforce [3, 4] Experiential training comprises acquisition of necessary knowledge, skills, competencies, attitudes and behaviors that enable a person to perform certain tasks adequately in their profession. Experiential training enables a professional to rapidly move from an awareness level in the proficiency of doing a task to being completely proficient, performing and teaching the task to others-in effect it is teaching and learning by doing. This concept of training has been adopted by a number of countries to build their public health workforces drawing experiences from the Epidemic Intelligence Service(EIS) which began training using this approach in United States of America in 1951[4–10]. This model has been adapted internationally to create the Field Epidemiology and Laboratory Training Program (FELTP) in several countries[1].

Nigeria adopted the experiential training approach to build its public health workforce in 2008 with the implementation of the multiagency Nigeria Field Epidemiology and Laboratory Training Program (NFELTP). This approach was embraced to augment other traditional training approaches by emphasizing a field based, competency-based approach to training public health workforce through a tiered approach. The training typically consists of a 2-year course leading to a master's degree in field epidemiology and public health laboratory management for midlevel public health leaders and competency-based short courses for frontline public health surveillance workers. Trainees and graduates work in multidisciplinary teams to conduct surveillance, outbreak investigations, and epidemiological studies for disease control locally and across borders.

The training is multi-sectoral and multi-displinary cutting across various cadres of health care workers and animal health sector professionals in the “one health” approach[11, 12]. NFELTP is a public health service-training program in applied epidemiology aimed at preparing leaders in field epidemiology to address public health issues and strengthen public health systems throughout Nigeria. The overall goals of NFELTP are: 1) to develop a self-sustaining institutionalized capacity to train public health leaders in field epidemiology (including veterinary epidemiology) and field-oriented public health laboratory practice and 2) to provide epidemiological services to the public health system at federal, state and local government levels. It is believed that a country would have an adequate coverage of public health workers trained in the experiential approach of the FELTP if there are three to five graduates of the program per million inhabitants working in suitable public health units [2].

The NFELTP is one of the premier FELTPs on the African continent. NFELTP was started as a joint effort between the Nigeria Federal Government (GON) through the Federal Ministry of Health (FMOH), the Federal Ministry of Agriculture and Rural Development (FMARD) and U.S. government through the Centers for Disease Control and Prevention (CDC). NFELTP aims to provide the country with the public health workforce that is needed to operate public health surveillance and response systems to implement the Integrated Disease Surveillance and Response (IDSR) strategy, address the Millennium Development Goals, implement the revised International Health Regulations and operationalize closer collaborations between the animal and human health (i.e., the “one world one health concept”). The experience of the avian influenza outbreak in Nigeria in 2006 demonstrated the importance of one health through the collaboration of the health sectors (human and animal) in capacity development and disease prevention and control. During and upon completion of training, NFELTP residents and graduates provide skill services to national and sub-national public health surveillance and response systems, with growing responsibility as they gain experience. With a population of 170 million, residing in 36 states and a Federal Capital Territory (FCT) and separated into six geopolitical regions there is a need to provide state and federal public health offices manned by professionals that have the ability to strengthen public health capacity to investigate and respond to outbreaks in addition to working together across disciplines collaboratively. To provide these services and work towards improving public health systems within Nigeria there is a need for the training of highly qualified individuals in field epidemiology to respond to the vast amount of public health concerns and threats that arise throughout the country. The different climatic patterns found in the three main geographic regions of the country: mangrove swamps to equatorial forest in the South, tropical in the Central, and Savannah in the North have implications for development of multiple public health challenges. For example the northern region is vulnerable to drought, desertification, food insecurity, and diseases especially cerebrospinal meningitis. In the south, disasters such as erosion, flooding and landslides, and vector-borne diseases are common. With recurrent infectious disease outbreaks, persistence of wild polio transmission, poor health outcomes, there is a need for investment in development of an effective customized and locally developed skilled public health workforce to address public health needs and priorities across the nation.

Weak surveillance systems coupled with untimely and uncoordinated response to disease outbreaks have continued to be a challenge in many African countries including Nigeria. Additionally, emerging pandemic threats require development of worldwide capacity for public health surveillance and response especially given the increased travel and urbanization. Good international public health surveillance and response, which is the basis of International Health Regulations (IHR) of 2005, cannot exist sustainably without good national surveillance and response operated by competent public health workforce in core public health positions at national and sub-national levels with a focus on disease detection, prevention and control. To achieve this, there is need to address several interrelated factors on human resources, disease surveillance and reporting capacity in an integrated and sustainable approach that enables the development of public health work force capacity in order to achieve public health surveillance and response systems that have a sustainable and adaptable capacity to address evolving public health needs[2].

This supplement is a demonstration of some of the results of NFELTP residents and graduates in addressing current public health challenges such as disease outbreaks and surveillance gaps for infectious and non-infectious diseases. The support to the program by the Government and its health development partners has enabled the program to upscale towards attaining its goal of building a public health workforce through experiential training and providing epidemiological services to improve public health in Nigeria and beyond. The authors describe the processes, operations and coverage of the program and provide disease specific examples on public health response. With the increasing appreciation of the need for global health security, NFELTP is laying the foundation for a new cadre of highly skilled public health workforce adaptable to numerous public health needs in a large diverse developing country beset with several health challenges. A skilled workforce is a prerequisite for strengthening national public health institutes and the program is creating the frontline health workers for this global initiative within the newly established Nigeria Centre for Disease Control. The authors in this supplement demonstrate the collaborative efforts of multiple agencies and the multi-disciplinary and multi-sectoral approach to optimal health of the population. With only six years of implementation and a newly developed 5 year strategic plan the program has shown that it can surmount its initial start-up challenges and play its rightful role in public health system strengthening[13].
